# A dynamic, multi-professional, needs-based simulation model to inform human resources for health planning

**DOI:** 10.1186/s12960-019-0376-2

**Published:** 2019-06-13

**Authors:** Adrian MacKenzie, Gail Tomblin Murphy, Rick Audas

**Affiliations:** 1 Division of Community Health and Humanities, Faculty of Medicine, Memorial University of Newfoundland, St. John’s, Canada; 2 WHO/PAHO Collaborating Centre on Health Workforce Planning and Research, Dalhousie University, Halifax, Canada; 3 Dunedin School of Medicine, University of Otago, Dunedin, New Zealand; 4 Nova Scotia Health Authority, Halifax, Canada

**Keywords:** HRH, Human resources for health, HHR, Health human resources, Health workforce, Planning, Health services, Multi-professional

## Abstract

**Background:**

As population health needs become more complex, addressing those needs increasingly requires the knowledge, skills, and judgment of multiple types of human resources for health (HRH) working interdependently. A growing emphasis on team-delivered health care is evident in several jurisdictions, including those in Canada. However, the most commonly used HRH planning models across Canada and other countries lack the capacity to plan for more than one type of HRH in an integrated manner. The purpose of this paper is to present a dynamic, multi-professional, needs-based simulation model to inform HRH planning and demonstrate the importance of two of its parameters—division of work and clinical focus—which have received comparatively little attention in HRH research to date.

**Methods:**

The model estimates HRH requirements by combining features of two previously published needs-based approaches to HRH planning—a dynamic approach designed to plan for a single type of HRH at a time and a multi-professional approach designed to compare HRH supply with requirements at a single point in time. The supplies of different types of HRH are estimated using a stock-and-flow approach.

**Results:**

The model makes explicit two planning parameters—the division of work across different types of HRH, and the degree of clinical focus among individual types of HRH—which have previously received little attention in the HRH literature. Examples of the impacts of these parameters on HRH planning scenarios are provided to illustrate how failure to account for them may over- or under-estimate the size of any gaps between the supply of and requirements for HRH.

**Conclusion:**

This paper presents a dynamic, multi-professional, needs-based simulation model which can be used to inform HRH planning in different contexts. To facilitate its application by readers, this includes the definition of each parameter and specification of the mathematical relationships between them.

## Background

Approaches to planning for human resources for health (HRH) can be categorized in several ways. A characteristic commonly used to distinguish between these approaches has been the means by which they estimated HRH requirements [1–9]. In this regard, HRH planning approaches fall into three main categories: supply-based (e.g., [10]), utilization- or demand-based (e.g., [11]), and needs-based approaches (e.g., [12]). Under supply-based approaches, requirements are estimated by multiplying current or target provider-population ratios by the estimated size of the future population. In utilization-based approaches, current or target utilization rates are multiplied by estimates of future population size which are then converted to HRH requirements using productivity estimates. Under needs-based approaches, best (or currently accepted) practices in terms of the number and type of services to be provided to individuals according to their level of health are applied to expected future distributions of levels of health across population groups, which are in turn applied to estimates of the future sizes of those groups. Provider requirements are then estimated from best practice (or current productivity norms) of rates of service provision per provider.

HRH planning approaches can also be classified in terms of the timeframes or professions they incorporate. Among the former categories, static models (e.g., [13, 14]) produce estimates of HRH supply and/or requirements at a single point in time, while dynamic models (e.g., [15, 16]) produce estimates for various future points in time, accounting for potential changes to planning parameters over time [17]. Among the latter categories, single-profession models estimate HRH supply and/or requirements for one type of HRH at a time (e.g., [18, 19]); they can also be applied to multiple professions independently (e.g., [20, 21]). In contrast, multi-professional models integrate planning for more than one type of HRH into a single model such that estimates of supply and/or requirements for each type of HRH are dependent on the others (e.g., [22, 23].

Two recent reviews across OECD countries found that HRH planning is almost invariably done on a profession-specific basis, without integration into broader health system planning or across professions [9, 24]. This is despite the fact that the increasingly complex nature of health care provision means that more and more individuals seeking care require the competencies of more than one type of health profession, and collaboration across these professions is increasingly required ([25], p. 26, [26–30]).

Static, multi-professional, needs-based HRH planning models have been developed for several contexts. Among the earliest of these was the health Need – service Target – Task – Productivity (NTTP) approach used by Kurowski and colleagues to plan for health workforces in Tanzania and Chad [31, 32]. In Australia, Andrews and the Tolkien II team developed a multi-professional needs-based approach to planning for mental health services [33]. Subsequently, Segal and colleagues, also in Australia, described multi-professional, needs-based planning for several different health conditions, including mental health conditions, in Australia [12, 14, 34, 35]. New Zealand’s Ministry of Health has undertaken workforce service forecasts for several different types of its services [36], including but not limited to youth health services [37] and mental health and addictions services [38], using this type of approach. Tomblin Murphy and colleagues have described the application of multi-professional, needs-based models to planning for older adults [39] and for pandemic influenza [23, 40] in Canada, and to planning for HIV/AIDS and malaria in Zambia [41].

In recent years, more sophisticated utilization- and demand-based approaches have also emerged that include measures of need as a determinant of HRH requirements and explicitly link HRH requirements to specific health care services. For example, Gallagher, Harper, and colleagues [22, 42, 43] have described a multi-professional, utilization-based HRH planning model for the dental workforce in England in which HRH requirements are determined as a function of population demographics, oral health status, expected patient attendance rates, and historical service utilization patterns among those patients. Most recently, Dall and colleagues have described a dynamic model to estimate demand for physician services in the United States that explicitly incorporates measures of population health as determinants of HRH requirements [44, 45].

As far as we have been able to determine, to date no HRH planning model has been presented in the scientific literature that is (1) dynamic, (2) multi-professional, and (3) needs-based. The purpose of this paper is to present such a model and demonstrate the importance of two of its parameters—division of work and clinical focus—which have not been included in previous models.

## Methods

In this section, the conceptual framework that guided the study is presented first, followed by the analytical framework specifying the relationships between the parameters included in the model is presented first. Next, the structure of the model is presented. Finally, a specific context used to demonstrate the model’s application is described.

### Conceptual framework

The study was guided by a conceptual framework for needs-based HRH planning developed by O’Brien-Pallas and colleagues [46]. The framework captures the fact that the supply of and requirements for HRH, as well as HRH planning itself, are influenced by an array of social, political, geographical, technological, and economic factors. Within this context, HRH planning begins by specifying the population health needs of the jurisdiction for which one is planning. Across all sectors of care, planning must work with the current stock of HRH, which is replenished by the production and recruitment of new providers. The flow of services from that stock is influenced by the level of resources allocated to it, the deployment of those resources, and the chosen model(s) of service delivery. These HRH, when supported by equipment and other non-human resources, yield patient, provider, and system outcomes that are optimized when there is an efficient mix of human and non-human resources in the jurisdiction.

### Analytical framework

The analytical framework on which this study’s quantitative methods are based has been adapted from the work of Birch and colleagues [47]. Under this framework, two distinct quantities are estimated and then compared over specified periods of time:

- The number of HRH available to deliver services to the population (supply); and
- The number of HRH required to deliver services to a population (requirements).

In this new model, HRH requirements are estimated using the following equation:

1 $$ {N}_{n,t}=\sum \limits_q\frac{\sum \limits_{h,i,j}\left({P}_{i,j,t}\times {H}_{h,i,j,t}\times {Q}_{h,i,j,q,t}\times {W}_{h,i,j,n,q,t}\right)}{R_{n,q,t}} $$

Where:

- *N*_*n,t*_ is the number of FTE HRH of type *n* required to deliver a given service model *Q*_*h,i,j,q,t*_ to a given population over a period of time *t*;
- *P*_*i,j,t*_ is the size of that population of age group *i* and gender *j* in the jurisdiction in question in time period *t* (i.e., demography);
- *H*_*h,i,j,t*_ is the proportion of the jurisdictional population with health status *h* of age group *i* and gender *j* in time period *t* (i.e., health status);
- *Q*_*h,i,j,q,t*_ is the mean number of services of type *q* planned or otherwise required, under a specified service model, to address the needs of individuals of health status *h* in age group *i* and gender *j* over time period *t* (i.e., level of service);
- *W*_*n,q,t*_ is the proportion of services of type *q* to be performed by HRH of type *n* for individuals of health status *h*, age group *i*, and gender *j* over time period *t* (i.e., division of work); and
- *R*_*n,q,t*_ is the mean number of services of type *q* that a full-time equivalent (FTE) HRH of type *n* can be expected to perform within time period *t* (i.e., productivity).

Different from the analytical framework originally described by Birch et al., specifying the division of work *W* according to the age, gender, and health status of individuals to be provided with each service allows for—but does not require—tailoring the planned service model for specific subpopulations and types of HRH. For example, this parameter can accommodate the specification that, for particularly sick patients, certain services will only be provided by specialist physicians. Because all the different types of services included in the level of service parameter (*Q*) must be allocated across professions through the division of work parameter (*W*), this parameter also makes explicit the interdependencies of requirements for individual types of HRH, because changing what is expected of one type of HRH automatically affects what is expected of the remaining types. For example, expanding the competencies and regulated scopes of practice of pharmacists to allow them to administer vaccinations means that, other things equal, nurses and physicians would be required to provide fewer vaccinations as pharmacists take on some of this workload.

The model estimates the supply of HRH using the following equation:

2 $$ {N}_{n,t}^{\prime }={S}_{n,t}\times {D}_{n,t}\times {A}_{n,t}\times {F}_{n,t} $$

Where:

- *S*_*n,t*_ is the number of HRH of type *n* qualified to practice in the jurisdiction during time period *t* (stock);
- *D*_*n,t*_ is the proportion of qualified HRH of type *n* who provide any direct patient care during time period *t* (participation);
- *A*_*n,t*_ is the mean proportion of an FTE devoted to direct patient care by participating HRH of type *n* during time period *t* (activity); and
- *F*_*n,t*_ is the mean proportion of an FTE devoted to the population(s) or health issue(s) being considered by HRH of type *n* during time period *t* (clinical focus).

The clinical focus term, which is not part of the framework described by Birch and colleagues, allows for explicit consideration of the proportion of providers’ time that is devoted to a specific type of clinical services. For example, it can be used to specify how much of primary health care nurse practitioners’ time is spent on patients’ mental health issues as opposed to respiratory illnesses, obstetrical care, musculoskeletal injuries, or other issues.

Other determinants of the supply of each included type of HRH are incorporated as shown in Eqs. (3)–(5):

3 $$ {S}_{n,t}=\sum \limits_i\left({S}_{i,n,t-1}\times \left(1-{E}_{i,n,t}\right)\right)+\sum \limits_i{I}_{i,n,t} $$

Where:

- *E*_*i,n,t*_ is the proportion of members of HRH of age group *i* and type *n* licensed to practice in year *t − 1* but did not retain their licenses for year *t* (e.g., due to retirement or migration to another jurisdiction);
- *I*_*i,n,t*_ is the number of members of HRH of age group *i* and type *n* entering practice in the jurisdiction in question in year *t*.

4 $$ {I}_{i,n,t}={G}_{i,n,t}\times \left(1-{O}_{n,t}\right)+{M}_{i,n,t} $$

Where:

- *G*_*i,n,t*_ is the number of HRH of age group *i* and type *n* who graduate from an entry-to-practice training program in the jurisdiction in question in year *t;*
- *O*_*n,t*_ is the proportion of new graduates of HRH of type *n* who do not begin practicing at least some direct patient care in the jurisdiction in question in year *t*; and
- *M*_*i,n,t*_ is the number of HRH of age group *i* and type *n* who obtain a new license to practice in the jurisdiction in question in year *t*.

5 $$ {G}_{i,n,t}=\sum \limits_y{C}_{n,t-y}\times \left(1-{F}_{n,t}\right)\times {D}_{i,t} $$

Where:

- *C*_*n,t − y*_ is the total number of students enrolled in all entry-to-practice training programs for HRH of type *n* in the jurisdiction in question in year *t* that are *y* years in duration;
- *F*_*n,t*_ is proportion of students in all entry-to-practice training programs in the jurisdiction in question who first enrolled in the program in year *y − t* and do not successfully complete it by year *t* (also referred to as “program attrition”); and
- *D*_*i,t*_ is the proportion of graduates of all entry-to-practice training programs in the jurisdiction in question of age group *i*.

Equations (3)–(5) illustrate how HRH supply is estimated using a stock-and-flow model ([26, 47–49], p., 55). In this case, the “stock” is the number of licensed care providers, the “flows” into that stock include new, locally trained graduates as well as those migrating in from other jurisdictions, and the flows out include those no longer holding a license to practice in the jurisdiction in question.

### Simulation model

**Fig. 1:**
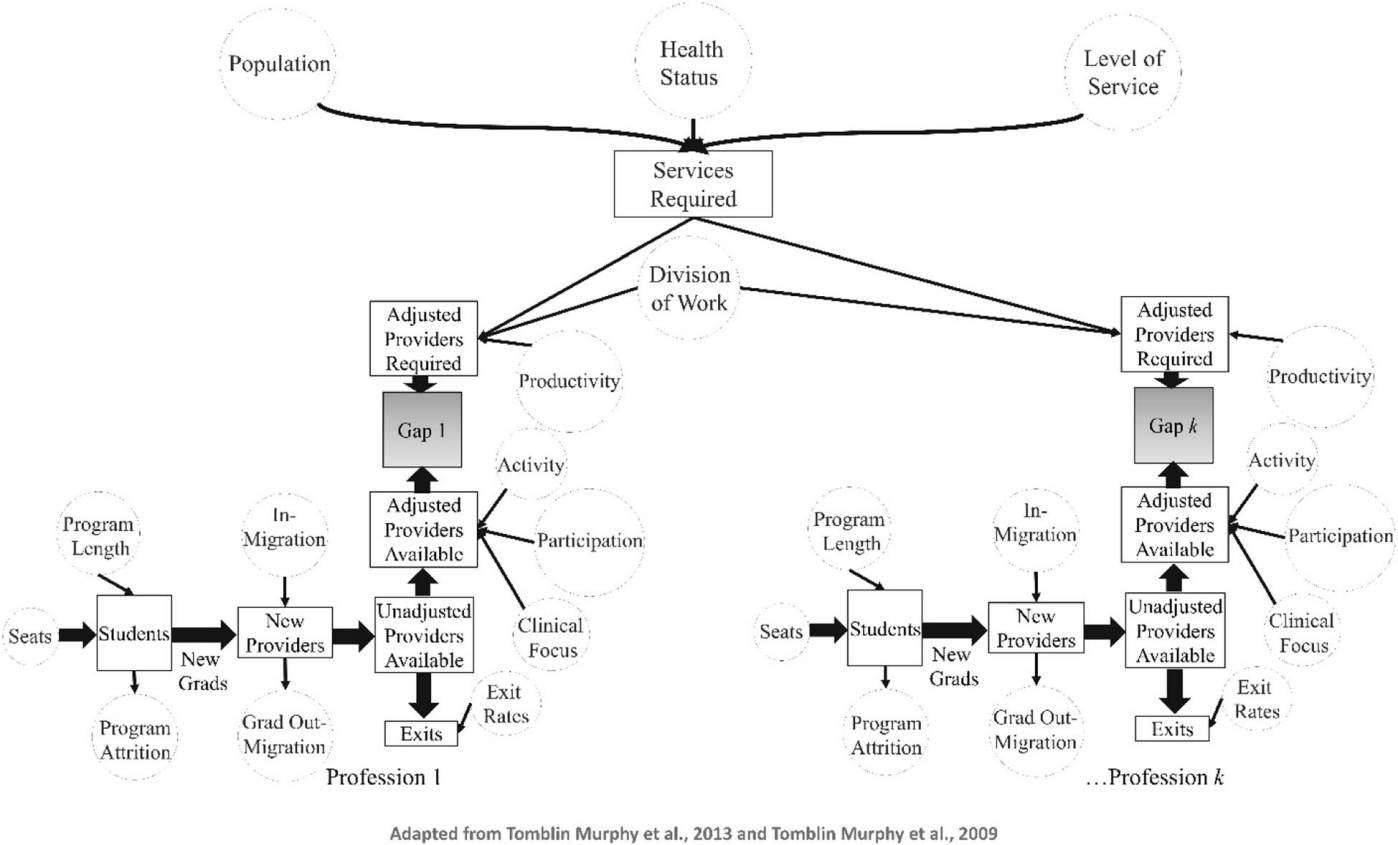
Visual representation of the model structure

The simulation modeling approach used in this study implements the analytical framework specified above and builds on both the dynamic, single-professional, needs-based simulation modeling approach [16, 18, 50, 51] and the static, multi-professional, needs-based planning approach [39, 41] developed by Tomblin Murphy and colleagues. A visual representation of the simulation model is shown in Fig. 1.

Like the dynamic model used by Tomblin Murphy and colleagues, the purpose of this approach is not to predict the future. Instead, the model intended to integrate knowledge of different types of HRH and other aspects of the health care system (such as planned service levels) into a single planning and communication tool so as to promote understanding of how various factors affect the supply of and/or requirements for HRH and identify policy levers for influencing these. Like approaches previously described by Tomblin Murphy and colleagues [16, 18, 50, 51], this approach is designed to enable health policy makers to “rehearse” potential policy changes by altering the value of the determinants in the model and then examining the estimated impacts of such changes on the supply of and/or requirements for given types of HRH. Ultimately, this simulation modeling approach is designed to help health policy and decision makers identify the most effective and efficient ways to manage HRH under different future scenarios.

The model components are defined as follows:

For each of *k* professions included in the model, the model estimates the flow of *new graduates* from the various pre-licensure educational programs for these professions based on the size of its enrollment (*seats*), the *program length* (in years), the proportion of entrants who graduate on time (*program attrition*), and the proportion of graduates who enter clinical practice within the region in which they graduated as opposed to migrating or entering administrative or other positions that do not include direct care provision (*grad out-migration*).

The model then estimates the future size of the stock of each included profession based on the current number of members of that profession currently licensed to practice (*unadjusted providers available*), the number of *new providers* entering that stock (either as *new graduates* or *in-migrants* from other jurisdictions), and the number of *exits* from that supply over time.

The unadjusted number of each type of providers available is then adjusted according to the proportion of licensed members who engage in at least some direct patient care (*participation*), the mean proportion of an FTE they devote to direct patient care (*activity*), and the proportion of that FTE they devote to the population and/or health conditions in question (*clinical focus*).

As specified in Eq. (1), the model estimates the number and type of services required based on the size and age-sex distribution of the population to be served (*population*), the distribution of health status within that population (*health status*), and the number and type of services to be provided according to different levels of health status (*level of service*). These are then translated into FTE requirements for different types of HRH by multiplying by the proportion of each type of service to be provided by each type of HRH (*division of work*) and dividing by the rate at which each type of HRH can be expected to perform each type of service (*productivity*).

Key differences between this model and those published previously, then, are the combined incorporation of:

- The *clinical focus* parameter;
- The *division of work* parameter; and
- Multiple parallel training and supply modules to account for dynamic changes to the supplies of *k* different types of HRH as opposed to a single type.

### Applied example

To demonstrate the impacts of making explicit the division of work and clinical focus parameters, the model described above was applied to planning for pediatric mental health services—specifically those pertaining to anxiety and depression among children aged 5–19—in the province of Nova Scotia, Canada.

Health care in Canada is largely administered through 13 publicly funded provincial and territorial insurance plans. Nova Scotia has a population of approximately 140 000 people aged 5–19. Publicly funded mental health services are mainly provided through two health authorities: the Nova Scotia Health Authority (NSHA), which administers the publicly funded health care system for most of the province, and the Izaak Walton Killam (IWK) Health Centre, which is located in the provincial capital of Halifax and is administered separately from the NSHA as it serves as the tertiary pediatric hospital for the neighboring provinces of New Brunswick and Prince Edward Island as well as Nova Scotia. Professions included in this example were:

- Nurses—including nurse practitioners (NPs) and registered nurses (RNs);
- Physicians—including family physicians (FPs), pediatricians, and psychiatrists;
- Psychologists; and
- Social workers.

Data to populate the model for the context of anxiety and depression among school-aged children were obtained from a combination of primary and secondary sources (Table 1). The latter included administrative databases of the provincial Department of Health and Wellness—specifically the provincial health insurance registry, physician billings database, and hospital discharge abstract database—as well as the regulatory bodies and professional associations of the included professions, the institutions in the province that provide the entry-to-practice education for these professions, and the scientific literature.

**Table 1:** Data availability by model parameter and profession

Model parameter	FPs/GPs	NPs	Pediatricians	Psychiatrists	Psychologists	RNs	Social workers
Population	Statistics Canada						
Health status	Asbridge et al., [52]; Meng & D’Arcy, [53]; Asbridge et al., [54]						
Level of service	UK Child and Adolescent Mental Health Services, 2017; Billings database; Clinician panel						
Existing stock	DHW	DHW	CIHI/DHW	CIHI/DHW	DHW	CIHI/DHW	NSCSW
Exit rates	DHW	DHW	DHW	DHW		CIHI	CIHI
Activity	Billings database	DHW	Billings database	Billings database	APNS	DHW	
Participation	DHW	DHW	CIHI/DHW	CIHI/DHW	APNS	CIHI/DHW	
Grad out-migration	CAPER/DHW	DHW	CIHI/DHW	CIHI/DHW		CIHI/DHW	
In-migration	CIHI/CAPER	DHW	CIHI/DHW	CIHI/DHW		CIHI/DHW	CIHI
Program attrition	CAPER	Program staff	CAPER	CAPER	Program staff	Program staff	Program staff
Program length	CAPER	Program staff	Program staff	Program staff	Program staff	Program staff	Program staff
Seats	CAPER	Program staff	CAPER	CAPER	Program staff	Program staff	Program staff
Division of work	Billings database	Clinician panel	Billings database	Billings database	Provincial role descriptions	Provincial role descriptions	Provincial role descriptions
	Clinician panel		Clinician panel	Clinician panel	Clinician panel	Clinician panel	Clinician panel
Productivity	Billings database	Clinician panel	Billings database	Billings database	Clinician panel	CIHI	
	Clinician panel		Clinician panel	Clinician panel		Clinician Panel	Clinician panel
Clinical focus	Billings database		Billings database	Billings database	APNS	DHW	NSCSW/ACCESS database

The former included a multidisciplinary panel of clinicians experienced in the treatment of anxiety and depression among school-aged children, which was convened to address gaps in the information available from other secondary sources. This approach has been used for similar reasons in previous HRH planning studies [40, 41].

## Results

This section begins with simulations of the future gap between the included types of HRH under a scenario in which all model parameters except population are held constant—referred to as a “status quo continues” scenario. Next, two pairs of scenarios related to the division of work and clinical focus parameters are presented—one of which shows how a change to that parameter can increase HRH gaps, the other of which shows how a change to that parameter can decrease HRH gaps. In each scenario presented here, the size and age-sex structure of Nova Scotia’s population of school-aged children is taken from Statistics Canada’s medium-growth projections based on Census data (Statistics Canada, 2017) [55].

### “Status quo continues” scenario

**Fig. 2:**
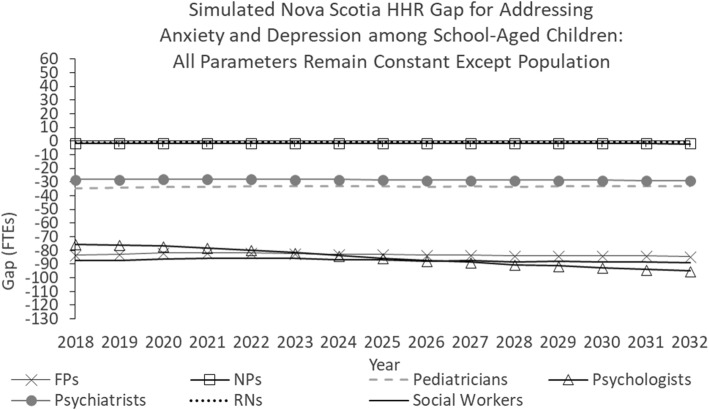
Simulated Nova Scotia HHR gap for addressing anxiety and depression among school-aged children: All parameters remain constant except population

Figure 2 shows the simulated Nova Scotia HRH gap for addressing anxiety and depression among school-aged children under a scenario in which all model parameters are held constant for the duration of the study period except population.

As Fig. 2 shows, as of 2018, there is a shortage of each type of HRH included in the model, including 83 FTE family physicians (FPs), 2 nurse practitioners (NPs), 35 pediatricians, 28 psychiatrists, 76 psychologists, 1 registered nurse (RN), and 87 social workers. In this scenario, the shortages of all professions but psychologists remain relatively stable throughout the simulation period, growing by 0–2 FTEs through 2032. The shortage of psychologists increases by 19 FTEs over the simulation period.

### Division of work scenarios

**Fig. 3:**
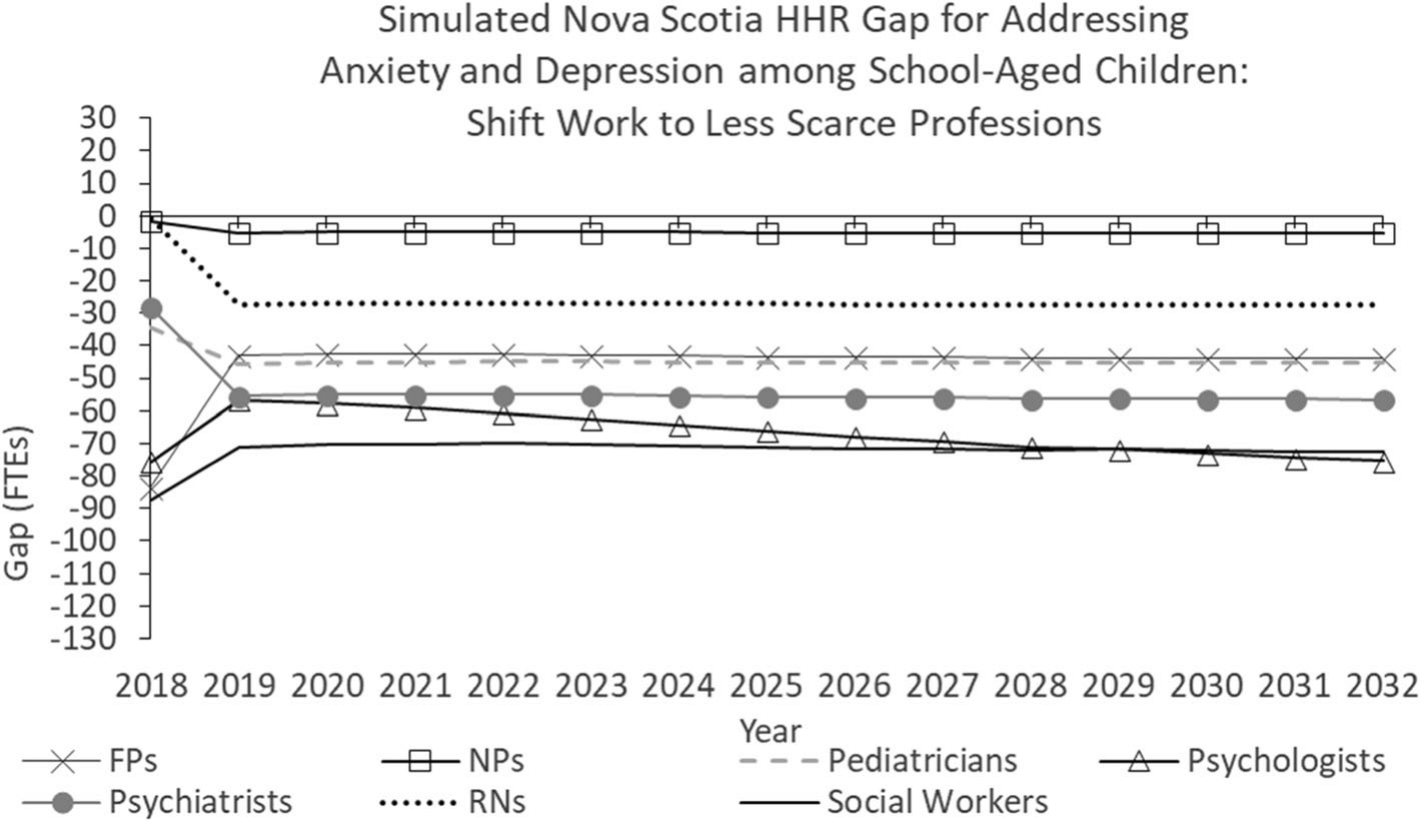
Simulated Nova Scotia HHR gap for addressing anxiety and depression among school-aged children: Shift work to less scarce professions

Figure 3 shows the simulated Nova Scotia HRH gap for addressing anxiety and depression among school-aged children under a scenario in which all model parameters are held constant for the duration of the study period except population and division of work. In this scenario, beginning in 2019, the division of work for several services are shifted from relatively “scarce” professions—physicians, psychologists, and social workers—to nurses, who are available in greater numbers.

More specifically, the following changes to division of work are used to illustrate their impacts:

- Instead of 60% of diagnostic assessments being performed by psychiatrists and 20% each by psychologists and social workers (as is specified in the “status quo continues” scenario), 10% are performed by family physicians, 5% by NPs, 10% by pediatricians, 40% by psychiatrists, 25% by psychologists, and 10% by social workers.
- RNs take on 40% of clinical assessments, reducing the proportions to be performed by FPs (from 15 to 10%), pediatricians (from 20 to 10%), psychiatrists (from 15 to 5%), psychologists (from 10 to 5%), and social workers (from 35 to 25%).
- Nurses take on 45% of care plan development (40% for RNs, 5% for NPs), reducing the proportions to be provided by FPs (from 10 to 5%), pediatricians (from 15 to 5%), and social workers (from 45 to 15%).
- Psychiatrists and pediatricians take on larger proportions (increasing from 1 and 9%, respectively, to 15% each) of individual psychotherapy sessions, reducing the proportions to be provided by FPs (from 30 to 15%) and psychologists (from 25 to 20%).

Each of the services listed above is within the respective scopes of practice of the various professions to whom they are being shifted [56]. These changes have the effect of reducing the simulated future shortages of family physicians, psychologists, psychiatrists, and social workers, and increasing those of NPs, pediatricians, and RNs.

**Fig. 4:**
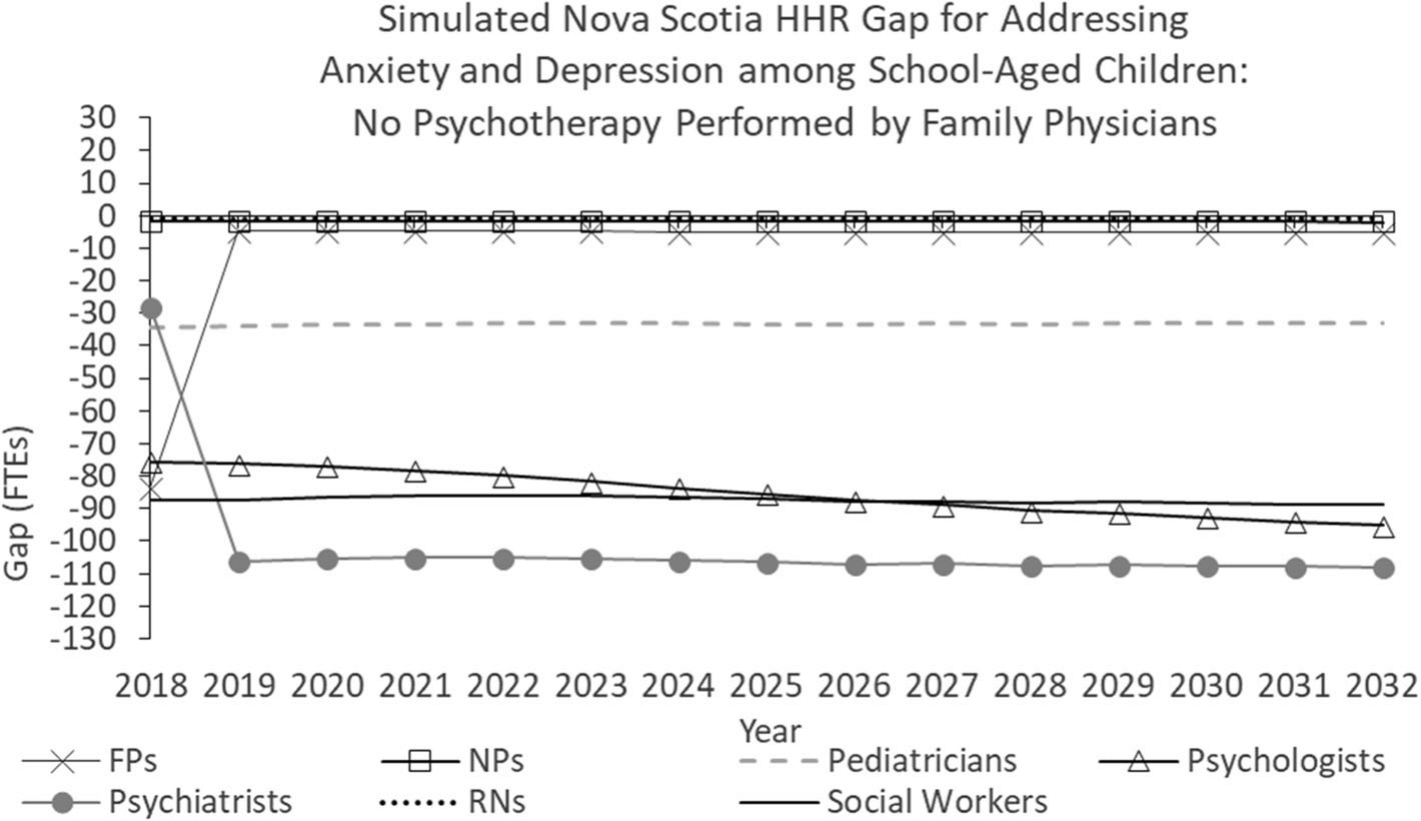
Simulated Nova Scotia HHR gap for addressing anxiety and depression among school-aged children: No psychotherapy performed by family physicians

Figure 4 shows the simulated Nova Scotia HRH gap for addressing anxiety and depression among school-aged children under an alternative scenario pertaining to the division of work parameter.

In this scenario, beginning in 2019, the psychotherapy services performed by family physicians in the baseline scenario are instead performed by psychiatrists. Such a policy change may conceivably be made to ensure these services are delivered by physicians with specialized training in mental health care. This has the effect of reducing the simulated future shortages of family physicians while increasing the simulated future shortage of psychiatrists. There is no effect on the other professions because the services they are expected to provide remain the same as in the “baseline” scenario.

### Clinical focus scenarios

**Fig. 5:**
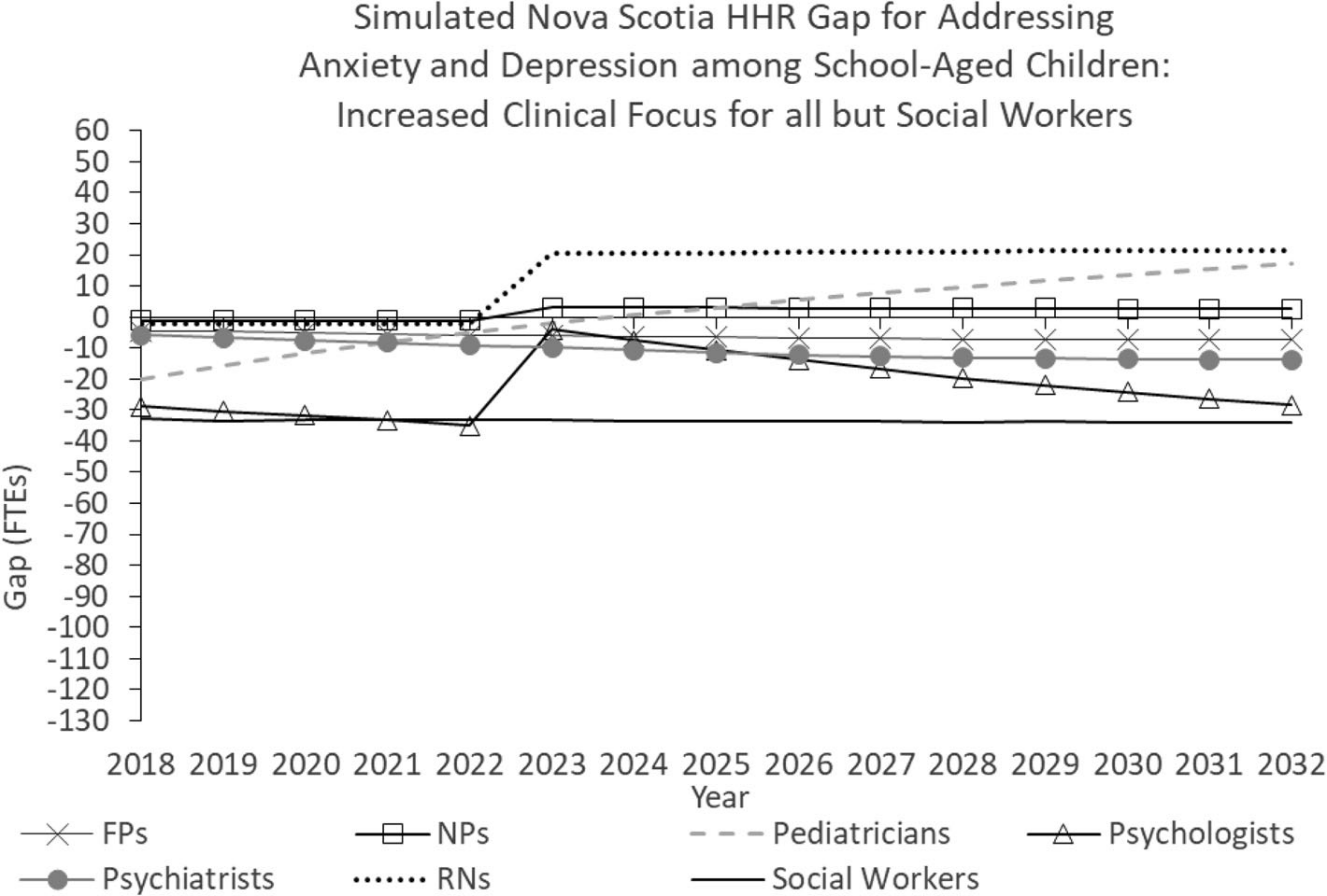
Simulated Nova Scotia HHR gap for addressing anxiety and depression among school-aged children: Increased clinical focus for all but social workers

Figure 5 shows the simulated Nova Scotia HRH gaps for addressing anxiety and depression among school-aged children under a scenario in which all model parameters are held constant for the duration of the study period except population and clinical focus. In this scenario, the clinical focus of all professions except social workers are increased so that these professions devote larger portions of their time to addressing anxiety and depression among school-aged children. More specifically, levels of clinical focus are increased from 0.4 to 2% for FPs, from 0.1 to 2% for NPs, from 1.8 to 10% for pediatricians, from 4.1 to 10% for psychiatrists, from 9.3 to 15% for psychologists, and from 0.1 to 1% for RNs.

The effect of these changes is to reduce the simulated future shortages of each of these professions. In the case of RNs, the simulated future shortage becomes a simulated future surplus.

**Fig. 6:**
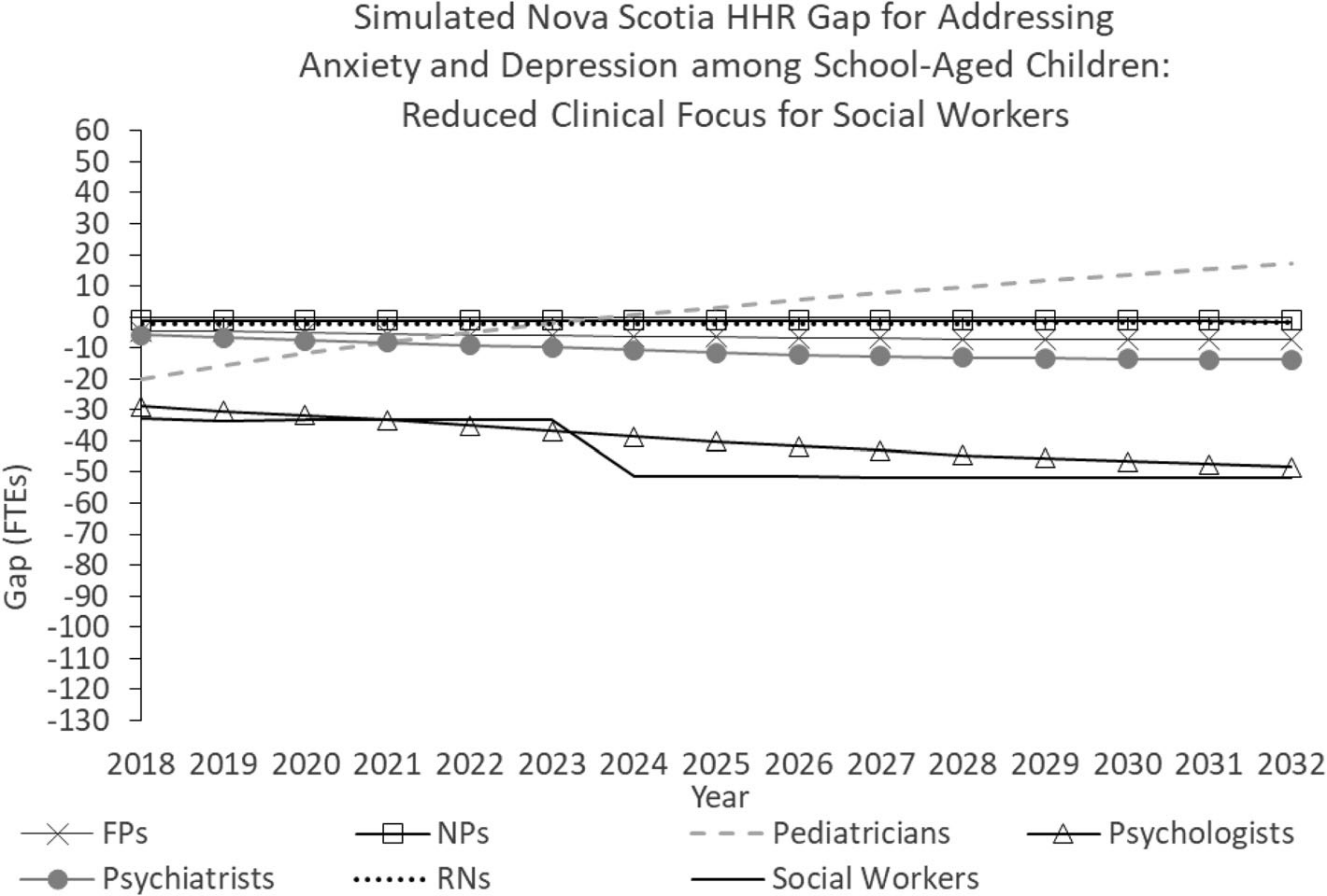
Simulated Nova Scotia HHR gap for addressing anxiety and depression among school-aged children: Reduced clinical focus for social workers

Figure 6 shows the simulated Nova Scotia HRH gap for addressing anxiety and depression among school-aged children under a scenario in which all model parameters are held constant for the duration of the study period except population and clinical focus. In this scenario, the clinical focus of social workers is reduced from 10 to 5% beginning in 2019. Such a policy change may be implemented, for example, to accommodate growing need for social workers’ services outside the health care sector [57].

This change increases the simulated future shortage of social workers from 89 to 107 FTEs compared to the “baseline” scenario. The simulated gaps for the other professions are not affected.

## Discussion

This paper provides the first known presentation of the mathematical structure of a dynamic, multi-professional, needs-based HRH planning model. Such models are important for HRH planning for several reasons.

First, dynamic approaches are needed to enable planners to anticipate the potential impacts of changes to planning parameters over time. This capacity is absent from, for example, the multi-professional, needs-based models described by Thomas, Konrad, and colleagues [58–60] and by Tomblin Murphy, Birch, and colleagues [39, 41].

Second, multi-professional models are needed because, as available health promotion strategies, and treatments and patients’ and families’ health care needs become more complex, addressing those needs increasingly requires the services of more than one type of health care provider. More broadly, health care is increasingly being provided through team-based approaches ([25], p., 26, [26, 28–30]). The capacity to incorporate planning for multiple professions in a single model is absent from, for example, the dynamic, needs-based models described by Tomblin Murphy, Birch, and colleagues [16, 18, 50, 51] or the dynamic, utilization-based models used by Health Workforce Australia [11].

Third, models that estimate health care service requirements as a function of population health needs (a) are consistent with the aim of addressing population health needs, which is shared by many health care systems [9], including Canada’s [61], and (b) allow for the explicit consideration of potential inefficiencies in the allocation of HRH relative to population health needs [51, 62–64].

In addition, needs-based HRH planning models are particularly important in Canada because their adoption is consistent with calls from multiple key health care stakeholder groups across the country to better align health goals, the health care system, and HRH planning with population health needs [65–74]. The capacity to account for differences in need for care and associated service requirements within populations and/or over time is absent from the HRH planning models most commonly used in OECD countries [9, 24].

Further, the example application of this model highlights the importance of two parameters—clinical focus and division of work—which have previously received little attention in the HRH planning literature. The potential of changing division of work to address HRH shortages has been the subject of much literature on “task-shifting”; however, there has been comparatively little attention given to factoring this concept into estimating HRH requirements. The closest examples are studies of planning for the oral health workforce described by Gallagher and colleagues [22, 42], and another on sexual, reproductive, maternal, newborn, and child health by ten Hoope-Bender and colleagues [76] building an approach described by the United Nations Population Fund (UNFPA) [75].

The former approach is technically demand-based as it estimates HRH requirements to meet the service needs of those who present for care (though it could be used for needs-based planning by assuming all those in need present for care); it also estimates future HRH supply using regression modeling as opposed to a stock-and-flow approach. The UNFPA model referenced in the latter two documents estimates current and future HRH requirements in a manner similar to that described in the present paper. It also estimates current and future HRH supply without distinguishing in-migration as a determinant of HRH supply; we have found no examples in the existing peer-reviewed literature of the potential of changing clinical focus to address HRH shortages in priority clinical areas being considered.

Nova Scotia’s health care professionals devote relatively little of their time to addressing the specific population and health issues of focus for this example—specifically anxiety and depression among school-aged children. For each of the seven professions included in the example, the estimated level of clinical focus on this population and these problems was less than 10%. For all but psychologists and social workers it was less than 5%. These low estimated levels of clinical focus are the primary reason that, for example, recently announced funding for additional family medicine residency seats [77] and additional capacity for Dalhousie University’s Nurse Practitioner program [78]—which have been incorporated into the analyses—has little impact on the simulated future HRH shortages pertaining to anxiety and depression among school-aged children.

The results pertaining to the division of work parameter highlight the potential impact of reassigning some health services from relatively scarce types of HRH—such as psychologists and psychiatrists—to less scarce types of HRH—such as RNs—that also have the competencies to provide those services. Such interventions can help to maximize the contributions of different types of HRH to addressing population health needs while also helping those HRH to work to their full scopes of practice.

### Limitations

Application of this planning approach is limited by the availability of relevant planning data to populate it. Available sources of data on population health status, levels of service provision, division of work, and levels of HRH productivity, activity, and clinical focus in Nova Scotia do not allow for these parameters to be estimated with precision. To address these gaps, existing data on these parameters were supplemented with estimates from the clinician panel and assumed values. As such, the results provided in this paper are intended to illustrate (a) the application of this approach overall and (b) the importance of the clinical focus and division of work parameters in particular.

As others have demonstrated [6, 7, 12, 24, 26, 42, 43, 51, 58, 79], the absence of systematically collected information on key health system and HRH planning parameters remains a problem for many health care systems, including those in developed countries. The results presented here illustrate the sensitivity of estimates of HRH supply and requirements, respectively, to values of clinical focus and division of work—yet it is rare that measures of these planning parameters are systematically captured in existing health care systems. In the present study, for example, billing data provided a systematic source of information on both parameters that is nonetheless subject to several important limitations, perhaps the most important of which is that these data do not measure the actual time physicians spend on different activities.

As Tomblin Murphy, Birch, and colleagues have repeatedly argued [9, 18, 80], problems with data are not avoided by adopting or reverting to the conceptually invalid models most commonly used by HRH planners worldwide, especially when these have been recognized as inadequate for decades [81–83] and have resulted in the shortages and inefficiencies in HRH allocation being experienced worldwide. The continual refinement of the application of a conceptually valid approach is superior to adopting conceptually invalid approaches based on the availability of data [18]. The methods and example application provided here represent one more addition to a growing evidence base established over decades demonstrating that reliance on fundamentally flawed traditional HRH planning models is unnecessary and counter-productive.

Applications of needs-based approaches in several developing countries where gaps in health system and HRH planning data are even more pronounced than they are in Canada [16, 31, 32, 41, 75, 76, 84–87] illustrate the potential of such approaches to advance HRH planning despite these gaps. The specific approach presented here has been designed so as to readily accommodate any more current or robustly collected data as these become available, so that if and when investments are made in addressing existing gaps in HRH and health system planning data—such as through the WHO’s National Health Workforce Accounts Program [88]—these can be reflected in the results of this approach.

## Conclusions

This paper presents a dynamic, multi-professional, needs-based simulation model which can be used to inform HRH planning in different contexts. To aid in its application by readers, this includes the definition of each parameter and specification of the mathematical relationships between them, as well as some sample results from its application to the real-world context of pediatric anxiety and depression among school-aged children in a Canadian province.

## Data Availability

The raw datasets used during the current study are not publicly available because they contain individuals’ personal information. The aggregate data used to produce the study results are available from the author upon reasonable request.

## Abbreviations

APNS: Association of Psychologists of Nova Scotia
CAPER: Canadian Post-MD Education Registry
CIHI: Canadian Institute for Health Information
DAD: Discharge Abstract Database
DHW: Nova Scotia Department of Health and Wellness
FP: Family physician
FTE: Full-time equivalent
GP: General practitioner
HRH: Human resources for health
IWK: Izaak Walton Killam Health Centre
LPN: Licensed practical nurse
NP: Nurse practitioner
NS: Nova Scotia
NSCSW: Nova Scotia College of Social Workers
NSHA: Nova Scotia Health Authority
OECD: Organisation for Economic Co-operation and Development
RN: Registered nurse
UNFPA: United Nations Population Fund
